# Medical Liability in Obstetrics/Gynecology and Co-liability With Anesthesiology in Greece: A Retrospective Study

**DOI:** 10.7759/cureus.30931

**Published:** 2022-10-31

**Authors:** Evangelia Samara, Lambros Tzoumas, Konstantinos Tzoumas, Minas Paschopoulos, Petros Tzimas, Georgios Papadopoulos

**Affiliations:** 1 Department of Anaesthesiology and Postoperative Intensive Care, Faculty of Medicine, School of Health Sciences, University of Ioannina, Ioannina, GRC; 2 Department of Anaesthesiology, University Hospital of Ioannina, Ioannina, GRC; 3 Department of Obstetrics and Gynaecology, Faculty of Medicine, School of Health Sciences, University of Ioannina, Ioannina, GRC

**Keywords:** obstetric anesthesiology, anesthesiology, litigation, medical liability, malpractice, obstetrics, gynecology

## Abstract

Objective

To evaluate the current landscape regarding medical liability in obstetric-gynecology (OB/GYN) physicians in Greece.

Materials and methods

Published court decisions of criminal, civil, administrative, and disciplinary content were searched in legal information banks for the years 1988-2021. The causes that led to the adverse outcome and the decisions were analyzed.

Results

A total of 184 decisions were directed against OB/GYNs. One hundred seventeen records concerned criminal cases and 67 civil cases. Thirty-four decisions concerned criminal cases of negligent homicide, 35 criminal cases of bodily harm, and 19 were acquittals. The most common causes of bodily injuries were neonatal encephalopathy, obstetric paralysis - quadriplegia and brachial plexus paralysis, and obstetric bleeding.

Conclusion

According to our results, there is a great need and challenge to maintain high standards in daily practice with continuous training and the use of international protocols. Furthermore, for each case, continuous monitoring of parturients and newborns and coordinated cooperation are necessary to reduce mortality and morbidity.

## Introduction

A surgical procedure has potential hazards not only for the patient but also for the surgeon and the anesthesiologist in case of a medical error [1-3]. Especially in obstetrics, there is a risk of bodily injury or death of both the mother and the fetus or newborn, with a potential claim of medical negligence. Maternal and perinatal deaths are unfortunate events for obstetricians globally and can be linked to negligence and litigation [1]. The potential threat of a legal dispute and possible monetary compensation is ever-present in everyday practice, and the monetary awards are often exorbitant [4]. It is worth noting that medical errors can occur regardless of the doctors’ experience. According to Vincent C et al., 75% of obstetricians and gynecologists practicing in the North Thames region, United Kingdom, had been involved in litigation. This has resulted in doctors, especially obstetricians, having one of the highest insurance rates compared to any medical specialty [1]. Closed claims analysis has been used previously to review risk patterns and to raise awareness. In addition, this methodology can suggest corrective or preventive action in future practice, thus minimizing the risk of future errors. Therefore, our research aimed to assess the current landscape regarding medical liability in obstetrics and gynecology in Greece, the reasons for the allegations of poor medical practice, and the relationship between these issues and the court results.

## Materials and methods

Published Greek court decisions of criminal, civil, administrative, and disciplinary content were searched from 1988 to 2021. Published court decisions were searched in the legal information banks Nomos, Sakkoulas online.gr and Bank of the Athens Bar Association, and in legal magazines, such as Nomiko Vima, Hellenic Justice, Criminal Chronicles, and Criminal Justice. The study excluded cases of death or encephalopathy due to general or regional anesthesia, which is part of another study [5].

The patients' age, operation dates, and causes of the adverse outcome were recorded. Court decisions were analyzed by an expert obstetrician and an anesthesiologist for the causes of death and the validity of the court decision in collaboration with the lawyers of the investigation. A determination was made as to the following: whether all patients underwent detailed history recording, continuous and urgent follow-up, whether all operations were performed in organized institutions, the prompt or delayed treatment, whether the obstetric bleeding treatment guidelines were applied, and the presence of safety rules violation. The investigation excluded all cases of bodily injury or death, in which the main responsibility should be sought from another specialty, such as anesthesiology.

## Results

A total of 600 court decisions were retrieved concerning surgeons and anesthesiologists, of which 184 (30.66%) concerned obstetric-gynecology (OB/GYNs), 103 (17.66%) general surgeons, and 84 (14%) anesthesiologists. The remaining 38% concerned orthopedics, neurosurgeons, cardiothoracic surgeons, vascular surgeons, plastic surgeons, otolaryngologists, and urologists.

One hundred seventeen records concerned criminal cases and 67 civil cases. Thirty-four decisions concerned criminal cases of negligent homicide, 35 criminal cases of bodily harm, and 19 were acquittals.

Convictions for manslaughter

Table 1 shows the age of parturients - patients who died due to alleged poor medical practice, the risk classification according to the American Society of Anesthesiologists (ASA), and the duration of the dispute (N = 30).

**Table 1:** Age of parturients - patients (excluding neonates) who died due to alleged poor medical practice, the risk classification according to ASA, and the duration of the dispute (Ν = 30). ASA: American Society of Anesthesiologists.

Mean age/years (SD), (range)	34 (10,6) (22-63)
ASA I-II	28
ASA III	2
Court dispute duration/years (SD), (range)	7.90 (2,15) (3-13)

In 19 criminal cases and in five civil cases, the OB/GYNs were convicted. Both the anesthesiologist and the OB/GYN were convicted in a total of 10 cases, five civil and five criminal. Table 2 summarizes the causes of homicide by negligence in criminal and civil cases.

**Table 2:** Causes of homicide by negligence in criminal and civil cases. * Conviction of both the Anesthesiologist and the Obstetrician-Gynecologist.

Cause	N
Hemorrhage	18
Uterine atony -- obstetric hemorrhage	6*
Placental abruption	3
Postoperative hemorrhage	3
Vaginal and cervical varicose vein rupture	1
Uterine rupture	3*
Cervical rupture	1
Uterine perforation	1
Neonatal death	4
Peritonitis	3
Misdiagnosis	2
Missed uterine cancer diagnosis.	1
Missed acute nephritis diagnosis.	1
Other	7
Allergic shock attributed to diclofenac.	1
Preeclampsia.	1
Anesthesia administration by the obstetrician to a heart disease patient.	1
Epidural anesthesia in a patient with a bicuspid, stenotic aortic valve.	1*
Tracheal perforation during intubation.	1 *
Fluid overload during laparoscopy	1*
Excessive narcotics dosage	1*

The monetary compensation awarded for the cases of homicide by negligence was EUR 100,000 to 320,000.

Conviction for exposure to reckless endangerment

In one case, the obstetrician was convicted for performing a cesarean section instead of a vaginal birth while intoxicated, followed by a life-threatening hemorrhage for both the mother and the fetus and surgical wound inflammation.

Conviction for bodily harm by negligence

There were 35 convictions for negligent bodily harm in 13 criminal and 22 civil cases. They concern 21 OB/GYNs, out of which four were trainees. Table 3 shows the causes of bodily harm due to negligence of the OB/GYN in criminal and civil cases and the amount of monetary compensation awarded. Cases for which the awarded compensation was not published included intra-operative and immediate postoperative complications, insufficient pregnancy follow-up, and cases of fetal brachial plexus paralysis. 

**Table 3:** Causes of bodily injuries due to negligence of the OB/GYN in criminal and civil cases and monetary award. *Monetary award for one of the cases OB/GYN: Obstetric-gynecology.

Cause	N	Monetary award (Euros)
Obstetric hemorrhage, vigil coma.	1	850,000
Patient encephalopathy.	2	100,000*
Perinatal asphyxia.	5	88,040*
Obstetric paralysis, tetraplegia.	3	75,000
Cystic fibrosis, inadequate parental information	1	500,000
Fibroid diagnosis instead of pregnancy.	1	500,000
Gauze forgotten.	2	50,000
Forgotten broken needle in breast	1	40,000
Damage post liposuction	1	40,000
Sepsis post cesarean section	1	3,234,400
Pelvic peritonitis post abrasion	1	300,000

Court decisions analysis

According to the court case analysis, the outcome of death and bodily injuries could have been avoided in 56 (81.16%) cases in which there was a delay in treatment and discontinuous monitoring, the guidelines were not followed, the operation was performed in doctor's office instead of a hospital, or a birth was assigned to a trainee. Table 4 shows the additional causes that contributed to the conviction for manslaughter and bodily harm by negligence. 

**Table 4:** Additional causes that contributed to the conviction for manslaughter and bodily harm by negligence.* *multiple causes for each case

Cause*	N=69 (%)
Lack of constant and efficient monitoring.	34 (49.3%)
Delayed treatment.	22 (31.9%)
Hemorrhage treatment guidelines violation.	15 (21.7%)
Negligence on hospital or ICU transfer	3 (4.3%)
Procedure held in an outpatient doctor’s office	2 (2.9%)
On call doctor not present.	2 (2.9%)
Incorrect administration of uterine contractors	3 (4.34%)
Unsupervised trainee.	2 (2.9%)

Acquittal

A total of 19 cases were those of acquittal (Table 5).

**Table 5:** Cases of acquittal decisions.

Case	N
Intrauterine fetal death.	5
Twin miscarriage 13 days post amniocentesis.	1
Undiagnosed VACTERL syndrome.	1
Baby born with cystic fibrosis.	1
Quadriplegia due to chromosomal anomaly	1
Internal bleeding post conical cervical resection.	1
Post bleeding emergency obstetric hysterectomy.	1
Maternal death 3 days post birth, due to pheochromocytoma.	1
Placenta previa, death due to obstetric hemorrhage.	1
Triplet gestation, shock, DIC.	1
Cesarean section, neonatal death due to undeveloped lungs	1
Gestational myocardiopathy, parturient’s death	1
Pelvic tumor resection, large bowel adhesions, death	1
Calf burn due to diathermy malfunction	1
Peritonitis not related to laparoscopy	1

In two cases of negligent homicide, there was a final suspension of criminal prosecution due to the statute of limitations. 1. Death of a parturient due to heart disease. 2. Death of a neonate.

## Discussion

In our research, more cases concerned OB/GYNs compared to the other surgical specialties in Greece [6-7]. The causes of death and bodily injury from poor medical practice varied widely, with bleeding and perinatal asphyxia due to misdiagnosis, delayed diagnosis, or inadequate care being the most common factors.

Maternal hemorrhage, neonatal mortality, and encephalopathy are global problems [1, 8-10]. Managing obstetric hemorrhage requires communication skills and teamwork. When the parturient does not respond to pharmacological treatment and immediate surgery, transfusion of blood products and concentrated coagulation factors (fibrinogen and prothrombin complex) are required [11-12]. According to Shevell T and Malone FD, reluctance to perform a hysterectomy in massive obstetric bleeding may be the most likely cause of death, unlike a lack of surgical principles or medical skills [13]. The use of mass transfusion protocols and clinical practice has been shown to improve bleeding management results [14].

Simulation can play an essential role in the training of the birth care team (obstetricians, anesthesiologists, and nurses), the coordinated management, and the development of safety and quality initiatives. Studies link the simulation training to the group's response to postpartum hemorrhage, to the effectiveness of performing emergency cesarean section to reduce neonatal mortality, to reduce neonatal cervical plexus injury, and to parturient trauma associated with forceps delivery [15].

In our research, the most common causes of bodily injuries were neonatal encephalopathy, obstetric paralysis, quadriplegia and brachial plexus paralysis due to delay from decision to childbirth and/or inadequate care. In contrast, parturient/patient encephalopathy was mostly attributed to obstetric bleeding or safety rules circumvention by the OB/GYN and the anesthesiologist. Our results are in line with the research of Deshpande SP et al., according to which parturient care was among the factors that influenced the threat to claims [16].

Substantial delay from a decision to childbirth and/or inadequate care is also presumed by Pierre F in most allegations of intra-abdominal fetal asphyxia or cerebral palsy. Most allegations concerning fetal asphyxia or cerebral palsy were based on abnormal fetal heart rate patterns and presumed excessive delay from the decision to delivery and/or inadequate care [17].

In the study by Berglund S et al. of 472 cases, 177 infants were considered to have suffered from severe suffocation due to poor childbirth practice. The most common malpractice cases related to childbirth concerned failure to monitor fetal well-being in 173 cases (98%), ignoring fetal asphyxia signs in 126 cases (71%), including careless use of oxytocin in 126 cases (71%), and the choice of a non-optimal mode of delivery in 92 (52%) cases [18]. According to Cohen, most allegations against OB/GYN are mainly related to labor and childbirth management. Although many of these cases accuse the OB/GYN of improper fetal monitoring during labor for signs of oxygen deprivation, in most cases, there is an underlying claim about making the right decisions and the time and manner of delivery [19]. Histological examination of the placenta plays an essential role in litigation to confirm sudden catastrophic events that occurred before or during childbirth or to detect occult thrombotic processes that affect fetal oxygenation, reduced reserve placental patterns, and adaptive responses to chronic hypoxia. It can also rule out intra-abdominal hypoxia by revealing certain histological patterns typical of acute chorioamnionitis and the inflammatory response of the fetus or compatible with metabolic diseases [20]. The death or bodily injury of the newborn was a topic of discussion in jurisprudence. According to one view, one exists from the moment that, regardless of the symptoms, the natural sequence of phases is set in motion, leading to the childbirth process. This position was followed (although not explicitly) by decisions 34/1981 and 1671/2003, 72/2008, and 132/2007 of the Council of Misdemeanor Courts of Rhodes. On the other hand, there is the view that one exists from the moment of exit from the mother's womb and only part of the newborn's body. This position was explicitly adopted by the Council of Misdemeanor Courts of Larisa, decision 74/2000, and the Council of Misdemeanor Courts of Athens, decision 1963/2013. The common denominator of both views, however, is that since the fetus is already dead in its mother's womb, there can be no question of bodily harm or death of a newborn. The question of when human life begins exists because any legal point of view is influenced by medical science and ethical-religious conceptions. In contrast, the penal code does not extend the protection of human life to a time before birth. In our investigation, most homicide or bodily harm convictions concerned obstetricians. In Habek D's research, in 63% of the cases of medical malpractice, the obstetrician was responsible; in 23.18% of the cases, both the obstetrician and the anesthesiologist; in 5.79%, the intensivist; in 4.34%, the gynecologist; and in 2.89%, the nurse/midwife [20].

The monetary compensations paid for bodily injury or death in the cases that we can find varied from 6,000 to 3,234,400 euros. In the research of Glaser LM et al., the average compensation was USD 279,384, and the procedure associated with the highest rate of paid claims was the vacuum extraction of the newborn [21]. However, financial compensation for poor medical practice by OB/GYNs often reaches exorbitant amounts [4]. In our investigation, there was an acquittal of an OB/GYN in 19 complaints as it did not prove to be medical malpractice on their part. In reality, however, they were punished as most of the defendants were subjected to many years of judicial harassment (average length of litigation was 8 years) resulting in mental ordeal, moral damage, wasted valuable time, reduced personality and significant financial burden, which looks like an indirect conviction in case of acquittal. The limitations of our study are that monetary awards were not published and accessible for all the cases, limiting the economic perspective and that there may be decisions for the timeframe researched that are not published yet.

## Conclusions

Our results show a great need and challenge in reducing maternal and neonatal mortality. Maintaining high standards in daily practice with continuous training and use of international protocols for each case, continuous monitoring of parturients and newborns, and coordinated cooperation can help further reduce mortality and morbidity.

In order to avoid an accusation of bad medical practice, there must be a signed informed consent of the patient or parturient and appropriate documentation of any procedure that is performed, as this can provide occupational safety to OB/GYN in case of medical appeal. During referral procedures, it is essential to have access to a legible cardiotocography (CTG), a well-documented partogram, a complete umbilical cord blood gas analysis, placental pathology, and an extensive clinical examination of the newborn.

Immediate judicial-legislative reform of the timeline for seeking any responsibility of physicians at every level (civil, criminal, administrative, disciplinary) is required in order to clear the relevant litigation quickly and with the appropriate means so that those involved are not subject to prolonged resolution.
